# The comprehensive DNA methylation landscape of hematopoietic stem cell development

**DOI:** 10.1038/s41421-021-00298-7

**Published:** 2021-09-21

**Authors:** Xianlong Li, Di Liu, Linlin Zhang, Haizhen Wang, Yunqiao Li, Zongcheng Li, Aibin He, Bing Liu, Jie Zhou, Fuchou Tang, Yu Lan

**Affiliations:** 1 State Key Laboratory of Proteomics, Academy of Military Medical Sciences, Academy of Military Sciences, Beijing, China; 2 Beijing Advanced Innovation Center for Genomics and Biomedical Institute for Pioneering Investigation via Convergence, College of Life Sciences, Peking University, Beijing, China; 3 Peking-Tsinghua Center for Life Sciences, Peking University, Beijing, China; 4 Key Laboratory for Regenerative Medicine of Ministry of Education, Institute of Hematology, School of Medicine, Jinan University, Guangzhou, Guangdong China; 5 State Key Laboratory of Experimental Hematology, Department of Hematology, Fifth Medical Center of Chinese PLA General Hospital, Beijing, China; 6 Ministry of Education Key Laboratory of Cell Proliferation and Differentiation, Beijing, China

**Keywords:** Haematopoietic stem cells, Epigenetic memory

Dear Editor,

Up to now, hematopoietic stem cells (HSCs) that contribute to all mature blood cell lineages in adults still cannot be efficiently regenerated in vitro, at least in part due to the insufficient understanding of their physiological developmental trajectories and the underlying precise regulatory mechanisms. In mouse embryos, the first HSCs are believed to be derived from a specialized arterial endothelial population named hemogenic endothelial cells (HECs) in the mid-gestational aorta-gonad-mesonephros region, and then via the intermediates of two types of pre-HSCs (CD45^−^ T1 pre-HSCs and CD45^+^ T2 pre-HSCs). The transient and dynamic process is termed endothelial-to-HSC transition. After that, newly emerged HSCs colonize the fetal liver (FL) for proliferation and then migrate to the bone marrow niche for lifelong hematopoiesis^1–3^.

Taking advantage of multidimensional single-cell technologies, the single functional HSC-primed HECs and pre-HSCs have been precisely captured, and the molecular regulatory network of the entire course of HSC development has been systemically revealed at the transcriptional level^2,4^. However, the role of epigenetic machinery orchestrating HSC fate commitment remains uncovered yet. Several lines of evidences have indicated that DNA methylation modification is essential for regulating the expression of master hematopoietic genes and has a crucial role in definitive hematopoiesis, for example, the demethylation of the promoters of several key transcription factors (TFs) including Runx1 and Spi1 induced by long non-coding RNA H19 initiates the hemogenic program of the embryonic endothelial cells to generate HSCs^5,6^. Nevertheless, the global DNA methylation dynamics of HSC development have not been revealed at the genome-scale.

To obtain a comprehensive DNA methylation landscape of HSC development, we isolated six functionally enriched continuous populations along the path of HSC development as we and others previously defined, namely arterial endothelial cells (AECs), HECs, T1 pre-HSCs, T2 pre-HSCs, FL HSCs, and adult HSCs^2,4^, and preformed 30-cell pool whole-genome bisulfite sequencing (WGBS) (Supplementary Tables S1, S2). The methylation characteristics formed distinct clusters associated with different developmental stages (Supplementary Fig. S1a). Although an overall high level and a similar distribution pattern of CpG methylation (mCG) in the genomic regions were observed across all cell types (Supplementary Fig. S1b, c), the specific changing dynamics did exist during HSC development. The average level of methylation was slight increased from AECs to HECs, and sharply decreased in T1 pre-HSCs, then gradually increased from T1 pre-HSCs to adult HSCs (Fig. 1a), which was not compatible with the dynamic expression patterns of Dnmt and Tet family genes, suggesting the involvement of complicated regulatory mechanisms in regulating the dynamics changes of methylation levels across different HSC development stages (Supplementary Fig. S1d). With the projection of the corresponding single-cell transcriptome data, the DNA methylation level and the transcriptional activity showed an obvious reverse trend from HEC onward (Fig. 1a), further suggesting a role of DNA methylation in orchestrating gene expression during HSC development.

**Fig. 1:**
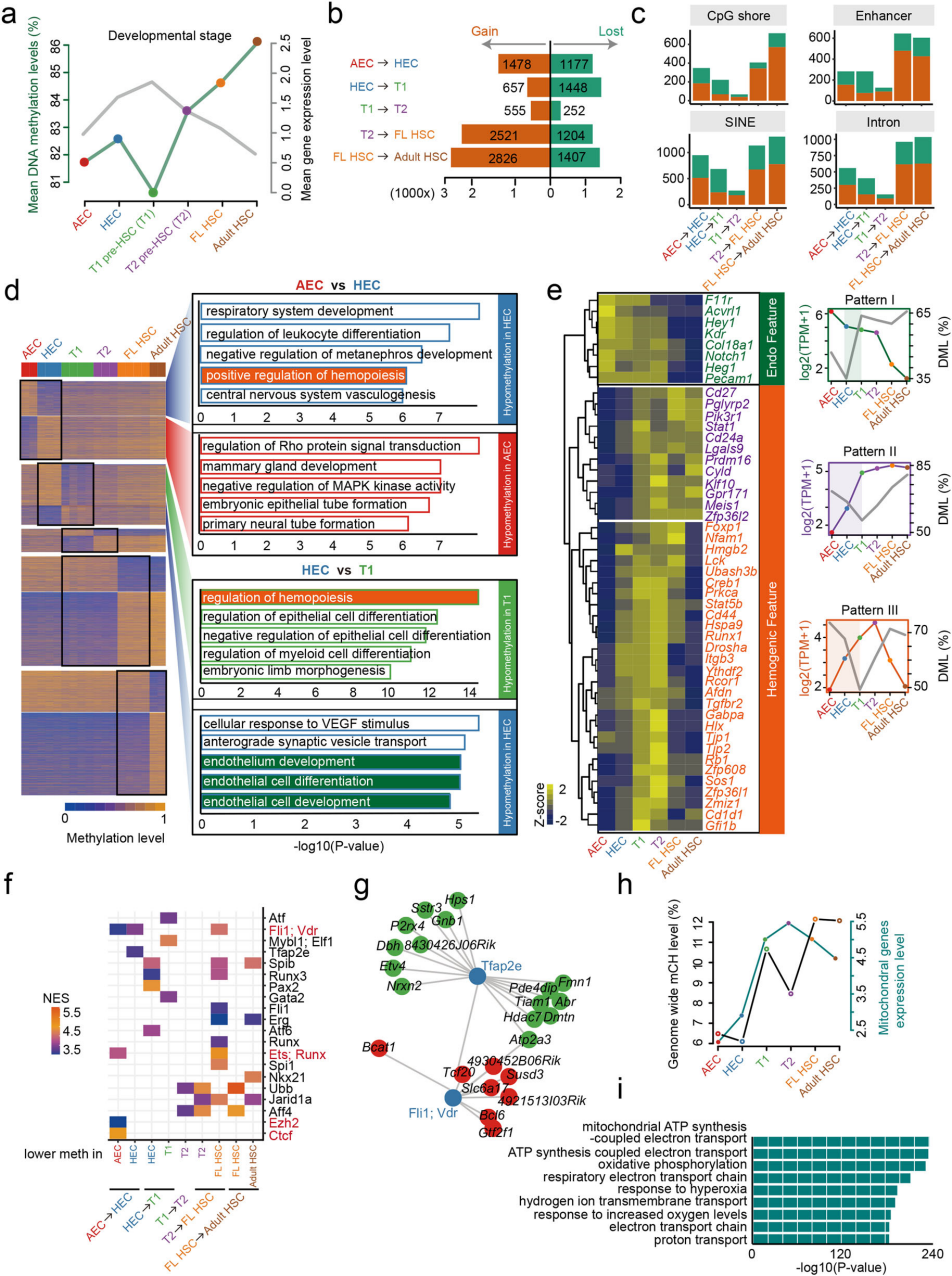
Dynamic DNA methylation landscape during HSC development. **a** Dynamic changes of DNA methylation level (mean) and RUVg normalized gene expression during HSC development. **b** The number of differential DNA methylation region (tiles 1k, cover base 5 bp, *P* value less than 0.01, the absolute value of differential methylation levels bigger than 25) was calculated between each two consecutive stages. **c** Histograms of the numbers of tiles with gain and lost DNA methylation between each two consecutive stages in the annotated genomic regions. **d** Heatmap of differential DNA methylation region between each two consecutive stages and Gene ontology (GO) analysis of DMRs by using GREAT and top five GO term are displayed. **e** Heatmap and hierarchical clustering of genes (row scale) from the highlighted GO term in **d** and three dynamic gene expression patterns and the corresponding methylation level displayed on the right side and highlighted with shadow. **f** Motif analysis of each gene-set (annotated from DMRs between each two consecutive stage) and normalized enrichment score bigger than 3.5 is showed. **g** Representative TF regulatory network was calculated based on iCisTarget. **h** Dynamic mCH level and mitochondrial genes expression level during HSC development. **i** GO analysis of mCH DMRs from HEC to T1 pre-HSC and top nine GO terms are shown.

To gain further insight into the mechanism of the DNA methylation dynamics, we identified totally 13,527 differentially methylated regions (DMRs) when compared between each two consecutive stages during HSC development (Supplementary Fig. S2a). The number of DMRs was the least in the course from T1 pre-HSCs to T2 pre-HSCs, indicating the developmental similarity of these two populations (Fig. 1b and Supplementary Fig. S2a). Notably, the numbers of loss-of-methylation regions were largely similar along the whole HSC developmental path except for that from T1 pre-HSCs to T2 pre-HSCs, while the high number of gain-of-methylation regions was associated with the two courses of colonization of HSCs, namely from T2 pre-HSCs to FL HSCs and the subsequent from FL to bone marrow HSCs (Fig. 1b). Interestingly, when analyzed the genomic distribution of these DMRs, we found the majority of them were enriched in intronic regions (such as short interspersed elements (SINEs) and introns) rather than other genomic regions during HSC development (Fig. 1c and Supplementary Fig. S2b), indicating that the dynamic methylation changes of these intronic regions might be involved in regulating gene expression by directly altering chromatin structures as previously reported^7,8^.

Next, the DMRs between each two consecutive stages were annotated to their nearby genes and the gene ontology (GO) analysis was performed by using the “Genomic Regions Enrichment of Annotations Tool” (GREAT). When compared with AECs, the loss-of-methylation DMRs in HECs was associated with the genes involved in specific terms of hematopoietic processes, such as “regulation of leukocyte differentiation” and “positive regulation of hemopoiesis”, suggesting that endowment of the hematopoietic fate in this stage was at least partially due to the reduced methylation on the locus of the hematopoietic genes. Interestingly, when compared with HECs, the loss-of-methylation DMRs in T1 pre-HSCs was further enriched in terms correlated to “regulation of hemopoiesis”, while the gain-of-methylation DMRs was significantly enriched in the terms associated with a variety of endothelium development processes. This finding indicated that the methylation changes on the locus nearby the endothelial and hematopoietic genes were clearly associated with the endothelial-to-hematopoietic fate switch that occurred during HEC to T1 pre-HSC transition (Fig. 1d and Supplementary Fig. S2c).

As one of the prominent roles of DNA methylation is to regulate gene expression, we further used our transcriptional profiling datasets^2,4^ to focus on the dynamic expression of the DMR-associated genes involved in specific endothelial or hematopoietic related GO terms highlighted in Fig. 1d and witnessed three different gene expression patterns along the whole course of HSC ontogeny. Expectedly, the expression level of the endothelial-featured genes showing gain-of-methylation in T1 pre-HSCs compared with HECs was gradually downregulated during HSC development, among which including arterial endothelial genes *Hey1* and *Notch1* in addition to pan-endothelial markers *Kdr* and *Pecam1* (Fig. 1e). On the other hand, the hematopoietic-featured genes with loss-of-methylation during endothelial-to-pre-HSC transition exhibited a consistent upregulation from AECs to pre-HSCs, which included the key TF *Runx1* (Fig. 1e). These results suggest that dynamic DNA methylation plays a role in negatively regulating the expression of a set of genes marking the stepwise processes of endothelial-to-pre-HSC transition.

Of note, the dynamic expression patterns of the genes did not persistently reversely correspond to their DNA methylation changes, such as that the expression of the endothelial-featured genes was dramatically decreased from T2 pre-HSCs to adult HSCs, whereas the corresponding DNA methylation remained largely unchanged (Fig. 1e, Pattern I). Moreover, two distinct expression patterns of the hematopoietic-featured genes were observed regarding their expression from T2 pre-HSCs onward, although their corresponding DNA methylation changes were similar. A subset of these genes (Pattern II) maintained high expression, including *Cd27*, *Cd24a*, and *Meis1*, whereas another subset (Pattern III) continuously downregulated expression thereafter, including *Cd44*, *Runx1*, and *Gfi1b* (Fig. 1e). The data suggest that distinct mechanisms might be involved in regulating the expression of these genes in different stages of HSC development.

Next, we mapped all DMRs to the canonical TF binding motifs and found that some of these TF motifs were significantly enriched in different populations (Fig. 1f). For example, when compared AECs with HECs, the Ets; Runx, Ezh2, and Ctcf recognized motifs were hypomethylated in AECs (Fig. 1f). The methylation changes of Ctcf recognized motifs suggest that CTCF-mediated chromatin remodeling might play a role in AEC to HEC fate switch^9^. In comparison, TF binding motifs for the Fli1; Vdr and Tfap2e were hypomethylated in HECs. A series of downstream target genes of Fli1; Vdr and Tfap2e were extracted, and several of them showed dynamic expression changes (Fig. 1g and Supplementary Fig. S2d). The finding suggests that the hypomethylation of these TF-recognized motifs were likely to be involved in regulating the expression of some of the presumed target genes of the TFs, and the role of which in hemogenic fate decision needs further experimental investigations.

Recently, several studies have revealed that the abundant non-CpG methylation (mCH) around the gene body was associated with transcriptional repression during fetal development^10^. Interestingly, the global mCH level was indeed significantly increased during HSC development (Fig. 1h), especially from HECs to T1 pre-HSCs. Further calculation and annotation of the mCH DMRs, we surprisingly found most of them were enriched in mitochondrial genomic regions and tended to upregulate the expression of mitochondrial genes involved in the regulation of energy metabolism (Fig. 1i and Supplementary Fig. S2e), which implies the unexpected role of mCH in regulating HSC development.

In summary, our study constructed a robust and biologically meaningful DNA methylation landscape of HSC development for the first time, and the specific DNA methylation patterns and their potential functions were predicted. However, due to the rare population of HSC-competent cells and their transient nature, the physiological function and precise regulatory mechanism of DNA methylation in HSC development needs further experimental verification by using more effective epigenetic intervention techniques in the future study. The comprehensive DNA methylation landscape should be a valuable resource for further exploring epigenetic mechanisms underlying HSC development and shedding new light on the strategies for directing HSC regeneration in the future.

## Supplementary information

Supplementary materials

## Data Availability

The scRNA-seq and WGBS data have been deposited in the NCBI Gene Expression Omnibus under accession number GSE153653 and GSE167237.
