# Supplementary material for: The comprehensive DNA methylation landscape of hematopoietic stem cell development
Source: Cell Discov. 2021 Sep 21;7:86. doi: 10.1038/s41421-021-00298-7 (PMC8452669; doi:10.1038/s41421-021-00298-7)
Supplement: Supplementary file 1 — Supplementary materials [file 41421_2021_298_MOESM1_ESM.pdf]

The comprehensive DNA methylation landscape of hematopoietic stem cell development

Supplemental information

The supplementary information consists of 2 supplementary figures, 2 supplementary tables, and materials and methods.

SUPPLEMENTARY FIGURES

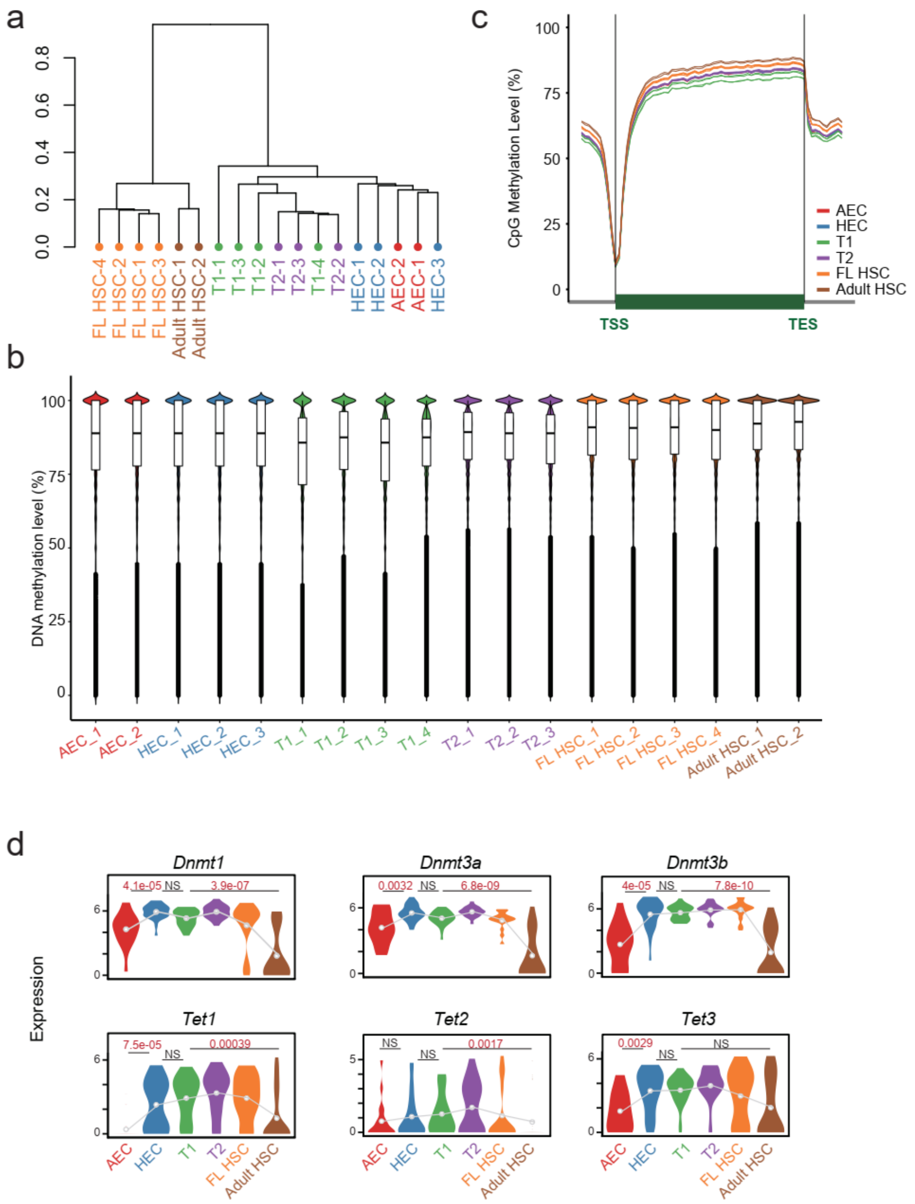

Supplementary Fig. S1 The global DNA methylation features during HSC development. (a) Hierarchical clustering using all samples. (b) Violin plot of global methylation level across each stage of HSC development. (c) Averaged DNA methylation levels along the gene bodies and 5 kb upstream of the transcription start sites(TSS) and 5 kb downstream of the transcription end sites (TES) of all RefSeq genes. (d) Violin plot of expression levels of DNA methylation-related enzymes across different HSC development stages, including DNA methyltransferases (*Dnmt1*, *Dnmt3a*, *Dnmt3b*) and methylcytosine dioxygenases (*Tet1*, *Tet2*, *Tet3*). Wilcoxon rank sum test is performed to evaluate the expression difference between the indicated stages.

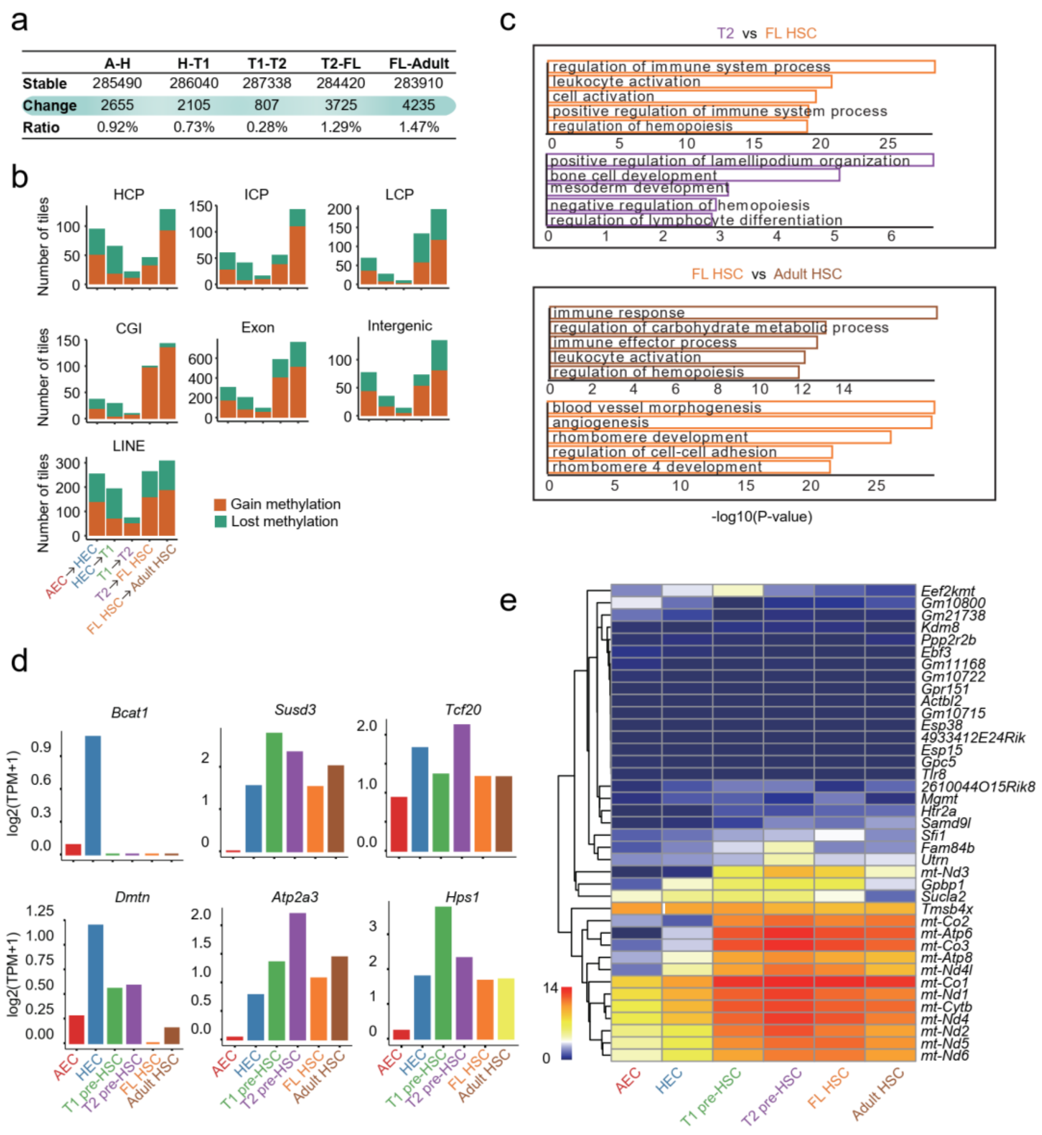

Supplementary Fig. S2 The DNA methylation dynamics of HSC development. (a) The numbers and ratios of changed DNA methylation regions between each two consecutive stages during HSC development. (b) Histograms of the numbers of gain or lost DNA methylation regions between each two consecutive stages in the annotated genomic regions. (c) Gene-ontology (GO) analysis of DMRs between T2 pre-HSC and E14 HSC, E14 and Adult HSC using GREAT. (d) The expression of several targeted genes regulated by the Fli1,Vdr complex or Tfap2e was shown. (e) Heatmap of the expression of genes annotated by increased mCH methylation region from HEC to T1 pre-HSC (mCH increased region defined as meth.diff > 1 and P-value < 0.05).

SUPPLEMENTARY TABLES

Supplementary Table S1 The FACS sorting strategy of indicated cell populations involved in this study.

Stage	Tissue	Cell_type	Surface Marker											
			CD31	CD41	CD43	CD44	CD45	c-Kit	CD201	Lin	Sca-1	Mac-1	CD150	CD48
E10.0	AGM	AEC	+	-	-	+	-	-						
E10.0	AGM	HEC	+	-		+	-	+	+					
E11.0	AGM	T1 pre-HSC	+	L			-	+	H					
E11.0	AGM	T2 pre-HSC	+				+	+	H					
E14.0	FL	FL HSC							+	-	+	L		
Adult	BM	Adult HSC					+		+				+	-

* E: embryonic day, FL: Fetal liver, BM: Bone Marrow, H: High, L: low

Supplementary Table S2 The information of sample quality control.

Sample	Mapping reads efficiency	Bisulfite conversation rate	Methylation calling
AEC-1	33.07%	99.10%	72.10%
AEC-2	32.67%	99.10%	72.00%
HEC-1	30.90%	99.10%	73.10%
HEC-2	31.72%	99.10%	72.50%
HEC-3	32.99%	99.10%	72.60%
T1 pre-HSC-1	23.81%	99.31%	58.91%
T1 pre-HSC-2	28.38%	99.32%	61.77%
T1 pre-HSC-3	27.72%	99.33%	60.54%
T1 pre-HSC-4	28.26%	99.27%	67.25%
T2 pre-HSC-1	30.61%	99.45%	69.59%
T2 pre-HSC-2	30.58%	99.43%	69.71%
T2 pre-HSC-3	26.95%	99.47%	67.94%
FL HSC-1	48.15%	99.13%	66.22%
FL HSC-2	45.02%	99.17%	66.76%
FL HSC-3	47.54%	99.14%	66.00%
FL HSC-4	45.62%	99.16%	65.38%
Adult HSC-1	39.72%	99.25%	65.43%
Adult HSC-2	36.05%	99.19%	69.95%

MATERIALS AND METHODS

Mice

C57BL/6 background mice were breed in Specific Pathogen Free (SPF) condition and mice experiments were approved by the ethics committee of the Laboratory Animal Center of Academy of Military Medical Sciences. E10.0 and E11.0 Embryos were staged by somite pair (sp) counting according to 31–35 sp and 41–45 sp separately. The caudal half or AGM region was then dissected into single-cell suspension by type I collagenase as previously reported[1].

Flow cytometry

Cells were sorted by flow cytometers BD Aria II, and the data were analyzed using FlowJo software (Tree Star). Cells were stained by the following antibodies: CD31 (BD or BioLegend, MEC13.3), CD41 (BD or eBioscience, MWReg30), CD43 (BD, S7), CD44 (eBioscience or BioLegend, IM7), CD45 (eBioscience, 30-F11), CD144 (eBioscience, eBioBV13), CD201 (eBioscience, eBio1560), Kit (eBioscience, 2B8), CD150(eBioscience, 9D1), CD48(eBioscience, HM48-1), 7-aminoactinomycin D (7-AAD; eBioscience) was used to exclude dead cells.

WGBS library preparation

The 30 cells WGBS libraries were constructed as previously reported with litter modification[2]. Briefly, bisulfite conversion was performed on cell lysates using MethylCode Bisulphite Kit (Invitrogen), after bisulphite conversion, the converted templates were conducted by the standard protocol. The final quality-insured libraries were quantified using Qubit ds DNA high sensitivity dye (Invitrogen) and then sequenced on NOVA PE 150 sequencer.

WGBS data alignment

For the WGBS data, we first remove the low-quality bases (less than 20) and trimmed adaptor sequences. Paired end reads were aligned to the mouse reference genome (mm19, downloaded from the UCSC genome browser) using Bismark tools (version 0.22.3) with default parameters[3]. The 48,502 bp lambda DNA genome was rebuilt as an extra reference for later calculating the bisulphite conversion rate of each sample.

Calculate the methylation levels of CpG and non-CpG sites.

We quantify the DNA methylation level of each sample, only the CpG sites with read coverage more than five times were taken into consideration, and we applied the 1000-bp-tile-based DNA methylation calling algorithm with methylKit[4]. For non-CpG methylation, considering they much lower level than CpG methylation level, we applied the 100-bp-tile-based DNA methylation calling algorithm to calculate average the methylation levels of all the non-CpG sites in each sample, only if they are covered more than ten times.

Identification of adjacent stage differentially methylated regions (DMRs).

We compared the DNA methylation levels of 1000-bp tiles between adjacent stages. DMR were calculated using methylKit[4]. We define the DMRs only if the difference methylation level of these tiles between adjacent stages bigger than 25% with a significant P-value less than 0.05 given by Chisq-test.

Mouse genomic region annotations

The genomic region annotations such as LINEs, SINEs, enhancers were downloaded from the UCSC genome browser. For other regions, such as CGIs, exons and introns were downloaded from UCSC tables with mm10 track. Intragenic regions were covered from TSS to TES, while the intergenic regions were defined as the complement of intragenic regions in the mouse genome. For each annotated genomic region, the DNA methylation level was calculated as the average DNA methylation levels of all CpG sites with the coverage more than five times.

Normalization of RNA-seq data

We use the single cell RNA-seq data from our previous research[5]. To quantify the average RNA expression level, RUV normalization algorithm[6] was employed for normalize RNA-seq data using ERCC spike-in controls. We scaled the log2(TPM+1) expression values of each gene among all the sample cells. For each stage, we averaged the scaled values of genes to show the dynamic change during HSC development.

GO analysis of DMRs

To study the influence of cis functions of DMR, we employed GREAT[7], which could incorporate distal binding sites and control false positives based on binomial test, for

Gene-ontology (GO) analysis of DMRs with default settings and "whole genome" as background.

Motif enrichment analysis

We annotated the DMR regions between adjacent stages to the nearest gene using annotatePeak function in ChIPseeker[8] with the parameters upstream from TSS 1kb and downstream from TSS 1kb. And we identify the significant enrichment motifs that are significantly over-represented in the surroundings of the TSS of the genes in the gene-set by calculate the area under the curve (AUC) for each pair of motif-gene Set with R package RcisTarget[9] to identify the significant enrichment motifs. The normalized Enrichment Score (NES) is calculated according to AUC distribution for each motif and the NES of the motif bigger than 3.5 is included for the following analysis. We choose i-cisTarget method to predict transcription factor regulatory network

1 Rybtsov S, Sobiesiak M, Taoudi S *et al.* Hierarchical organization and early hematopoietic specification of the developing HSC lineage in the AGM region. *J Exp Med* 2011; **208**:1305-1315.

2 Smallwood SA, Lee HJ, Angermueller C *et al.* Single-cell genome-wide bisulfite sequencing for assessing epigenetic heterogeneity. *Nat Methods* 2014; **11**:817-820.

3 Krueger F, Andrews SR. Bismark: a flexible aligner and methylation caller for Bisulfite-Seq applications. *Bioinformatics* 2011; **27**:1571-1572.

4 Akalin A, Kormaksson M, Li S *et al.* methylKit: a comprehensive R package for the analysis of genome-wide DNA methylation profiles. *Genome Biol* 2012; **13**:R87.

5 Zhou F, Li X, Wang W *et al.* Tracing haematopoietic stem cell formation at single-cell resolution. *Nature* 2016; **533**:487-492.

6 Risso D, Ngai J, Speed TP, Dudoit S. Normalization of RNA-seq data using factor analysis of control genes or samples. *Nat Biotechnol* 2014; **32**:896-902.

7 McLean CY, Bristor D, Hiller M *et al.* GREAT improves functional interpretation of cis-regulatory regions. *Nat Biotechnol* 2010; **28**:495-501.

8 Yu G, Wang LG, He QY. ChIPseeker: an R/Bioconductor package for ChIP peak annotation, comparison and visualization. *Bioinformatics* 2015; **31**:2382-2383.

9 Aibar S, Gonzalez-Blas CB, Moerman T *et al.* SCENIC: single-cell regulatory network inference and clustering. *Nat Methods* 2017; **14**:1083-1086.
